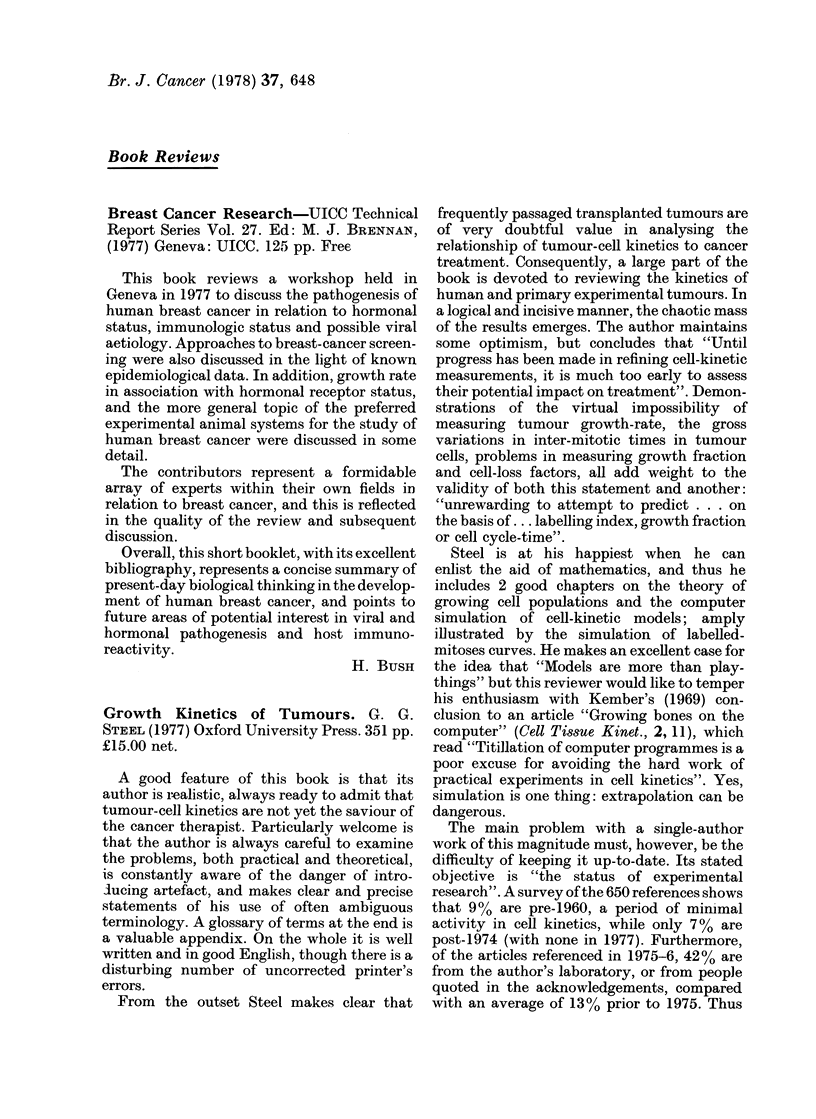# Breast Cancer Research

**Published:** 1978-04

**Authors:** H. Bush


*Br. J. Cancer* (1978) **37**, 648

### Book Reviews

**Breast Cancer Research**—UICC Technical Report Series Vol. 27. Ed: M. J. Brennan, (1977) Geneva: UICC. 125 pp. Free

This book reviews a workshop held in Geneva in 1977 to discuss the pathogenesis of human breast cancer in relation to hormonal status, immunologic status and possible viral aetiology. Approaches to breast-cancer screening were also discussed in the light of known epidemiological data. In addition, growth rate in association with hormonal receptor status, and the more general topic of the preferred experimental animal systems for the study of human breast cancer were discussed in some detail.

The contributors represent a formidable array of experts within their own fields in relation to breast cancer, and this is reflected in the quality of the review and subsequent discussion.

Overall, this short booklet, with its excellent bibliography, represents a concise summary of present-day biological thinking in the development of human breast cancer, and points to future areas of potential interest in viral and hormonal pathogenesis and host immunoreactivity.

H. Bush